# Fiber Optic Sensing Technology and Vision Sensing Technology for Structural Health Monitoring

**DOI:** 10.3390/s23094334

**Published:** 2023-04-27

**Authors:** Haojie Wang, Jin-Kun Guo, Han Mo, Xikang Zhou, Yiping Han

**Affiliations:** 1 School of Physics, Xidian University, Xi’an 710071, China; 2 School of Optoelectronic Engineering, Xidian University, Xi’an 710071, China

**Keywords:** fiber optic sensing technology, vision sensing technology, integration, structural health monitoring, SHM

## Abstract

Structural health monitoring is currently a crucial measure for the analysis of structural safety. As a structural asset management approach, it can provide a cost-effective measure and has been used successfully in a variety of structures. In recent years, the development of fiber optic sensing technology and vision sensing technology has led to further advances in structural health monitoring. This paper focuses on the basic principles, recent advances, and current status of applications of these two sensing technologies. It provides the reader with a broad review of the literature. It introduces the advantages, limitations, and future directions of these two sensing technologies. In addition, the main contribution of this paper is that the integration of fiber optic sensing technology and vision sensing technology is discussed. This paper demonstrates the feasibility and application potential of this integration by citing numerous examples. The conclusions show that this new integrated sensing technology can effectively utilize the advantages of both fields.

## 1. Introduction

The rapid development of various industries in today’s society has caused the composition, form, and function of many structures to become increasingly complex [1]. Especially since the 1990s, there has been an increasing emphasis on monitoring the quality and longevity of modern structures, due to the implementation of engineering projects such as large concrete buildings, large steel structures, and geotechnical structures. Long-term exposure to complex working conditions will inevitably result in varying degrees of damage and defects in these structures [2]. These potential damages and defects will make a structure much less stable and safe, thus increasing the likelihood of catastrophic accidents.

If these damages and defects are not foreseen and monitored in a timely manner, they can not only cause significant property damage but also pose a great threat to personal safety. Therefore, there is a great need for real-time health monitoring and assessment of structures to improve their safety performance. Structural health monitoring (SHM) is a technology that can meet this need very well, and it has become a popular topic of research in the engineering and scientific community worldwide [3,4].

**Figure 1:**
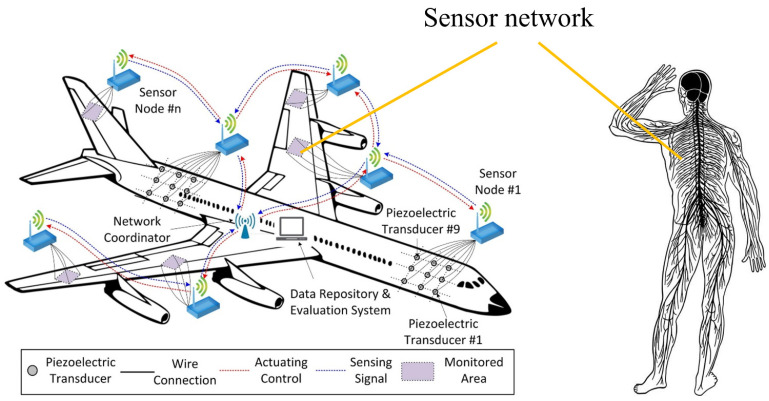
SHM as an imitation of the human nervous system (adapted from [9]).

The concept of SHM was first proposed and researched within the field of aviation [5], and it is based on the idea of mimicking the human nervous system [6], as shown in Figure 1. The SHM system consists of sensors, data acquisition and transmission systems, structural warning and assessment systems, and data management systems [7]. It uses sensors integrated into the structure to obtain information related to the health of the structure, such as strain and temperature, in real-time. It then uses a transmission system to store this information in a data management system. Finally, it processes this information using a structural warning and assessment system to obtain the health status of the structure. Thus, the sensors are the crucial tools by which the SHM system obtains information, and are considered the sensory organs of the SHM system [8]. Therefore, the implementation of SHM systems relies heavily on the support of sensing technology.

Fortunately, with the research on information technology, various sensing technologies have become increasingly mature [10]. A variety of sensing technologies have been used for structural health monitoring, such as piezoelectric elements [11,12,13,14], strain elements [15,16,17,18], ultrasonic sensing technology [19], and optical sensing technology [20,21,22,23]. Among them, due to its unique advantages in recent years, optical sensing technology has achieved rapid development and application around the world [24,25]. The optical sensing technology introduced in this paper is not only the widely used fiber optic sensing technology (FOS), but also vision sensing technology, which has become popular in recent years. In addition, we describe their future directions, including the integration of fiber optic sensing technology with vision sensing technology. In addition, we analyze the feasibility and advantages of this hybrid sensing system by describing some existing work. This paper is organized as follows: Section 2 introduces the basics and principles of FOS, Section 3 reviews the progress of FOS, Section 4 describes the applications of FOS, Section 5 reviews vision sensing technology, Section 6 provides future directions for optical sensing technology in SHM applications, and Section 7 summarizes the work in this paper.

## 2. Basics and Principles of Fiber Optic Sensing Technology

### 2.1. Basics of Fiber Optic Sensing Technology

FOS emerged in the 1970s along with the development of fiber optic technology and fiber optic communication technology [26]. These technologies use optical fiber as the transmission medium for information, and light as the information carrier. Compared with traditional sensors, FOS has many unique advantages [19,27,28], such as small size and light weight, fast response time, high security, good multiplexing and easy networking, a large bandwidth and high sensitivity, adaptability, anti-electromagnetic interference (EMI), anti-environmental interference, and so on. Therefore, since 1989, when Mendez et al. [29] first proposed the application of fiber optic sensors to concrete structure monitoring, FOS has quickly developed into a widely used sensor type in SHM systems.

**Figure 2:**
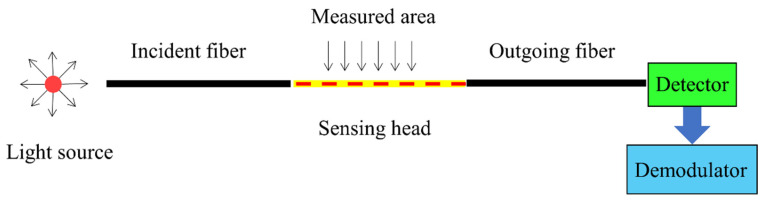
Schematic diagram of the working principle of FOS.

The FOS system includes five parts [30]: a light source, optical fiber, sensing head, photodetector and demodulator. As shown in Figure 2, its basic principle is to input light from a light source into an optical fiber and transmit it through the fiber to the sensing head. Inside the sensing head, the externally measured parameters interact with the light, causing the optical properties of the light, such as the intensity, phase, frequency, wavelength, polarization, etc., to change. This modulated signal light is then sent through the optical fiber to the photodetector and demodulator, and we finally obtain the measured parameters.

**Figure 3:**
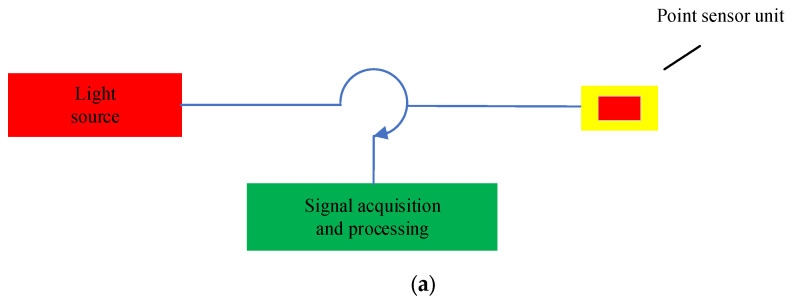

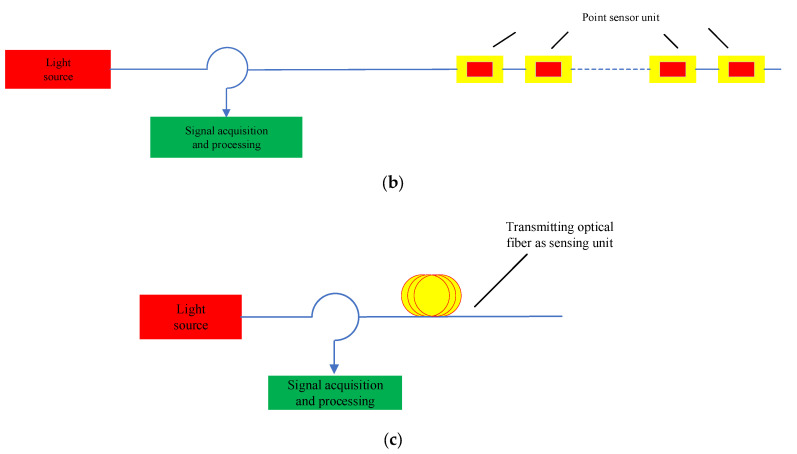
Schematics of fiber optic sensors. (**a**) single-point fiber optic sensors; (**b**) quasi-distributed fiber optic sensors; (**c**) distributed fiber optic sensors.

There are many ways to classify FOS. For example, according to the above, the commonly used optical modulation principle can be divided into intensity modulation, phase modulation, frequency modulation, wavelength modulation and polarization modulation FOS. By the role of optical fiber in the sensor, they can be divided into functional and non-functional FOS. According to the physical quantity to be measured, they can be divided into pressure, temperature, strain, and other FOS. In this paper, we introduce FOS according to their operating modes, which are divided into point-type FOS and distributed FOS (DFOS). Point-type FOS is also known as discrete FOS, and it is also referred to as local FOS [31]. If the size of the sensing element is much smaller than the size of the structural part, it is a point-type FOS. Due to the different numbers of sensor units, point-type FOS are further divided into single-point FOS and multi-point FOS (quasi-distributed fiber optic sensors) [32]. The DFOS on which each point is a sensing unit is a continuously distributed measuring instrument [33]. The respective schematics of these types are displayed in Figure 3 [34]. Of course, this classification cannot include all FOS, nor can the content of this paper include all FOS. Only some typical FOS are introduced and analyzed in the following sections.

### 2.2. Principles of Fiber Optic Sensing Technology

#### 2.2.1. Extrinsic Fabry–Perot Interferometer Sensors

**Figure 4:**
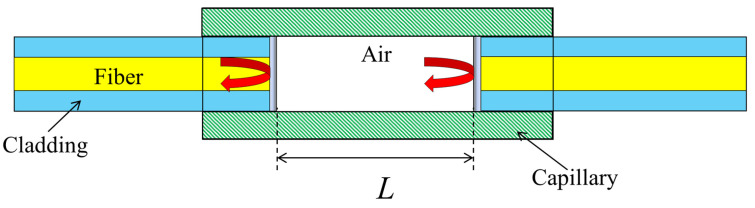
The structure of the EFPI sensors.

Extrinsic Fabry–Perot Interferometer (EFPI) sensors are based on multi-beam interference to achieve measurements. In them, the beams between the two mirrors interfere after multiple reflections [35], as shown in Figure 4. Let the intensity of the incident light be $I_{0}$, and the intensity of the interfering light after multiple reflections be I. The relationship between them is as follows [24]:

(1) $$\frac{I}{I_{0}}=2R\left[1+\cos\left(\frac{4\pi n_{0}L}{\lambda }\right)\right]$$

where L is the length of the cavity, λ is the wavelength of the incident light, $n_{0}$ is the refractive index of air, and R is the interface reflectivity. When the sensor is subjected to longitudinal stress with a strain, the cavity length L becomes L+ΔL=L+εL [36], and the interfered light intensity I becomes I+ΔI. The ratio of Equation (1) becomes the following [34]:

(2) $$\frac{I+\Delta I}{I_{0}}=2R\left\{1+\cos\left[\frac{4\pi n_{0}}{\lambda }L\left(1+\varepsilon \right)\right]\right\}$$

It can be seen that the strain causes the interference light to shift in phase. The phase shift can be decoupled through the detection of changes in the intensity of the reflected light, and then the strain on the EFPI sensors can be calculated.

#### 2.2.2. Fiber Bragg Grating Sensors

**Figure 5:**
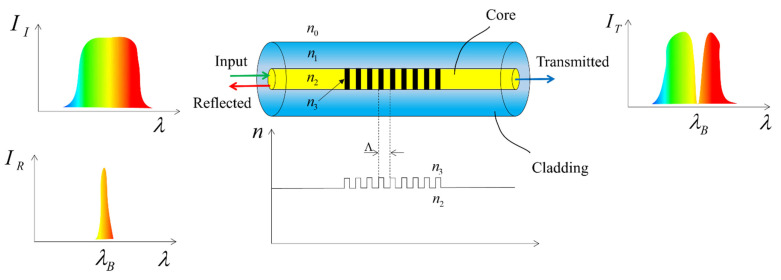
Schematic of the working principle of FBG.

Fiber Bragg Grating (FBG) sensors are functional fiber optic sensors that use fiber Bragg gratings as sensitive elements. They can be used to measure temperature and strain. As shown in Figure 5, when a beam of different wavelengths (shown in different colors in the figure) passes through the FBG, reflected light of a specific wavelength is generated. The relationship between the peak Bragg reflection wavelength $\lambda_{B}$ and the fiber grating period Λ is as follows [37]:

(3) $$\lambda_{B}=2n_{eff}\Lambda$$

where $n_{eff}$ is the effective refractive index of the fiber.

From Equation (3) above, we can see that the $\lambda_{B}$ of FBG depends on the $n_{eff}$ and Λ of the fiber. Any physical process that causes a change in these two parameters will cause a shift in $\lambda_{B}$. By detecting the shift in $\lambda_{B}$, it is possible to obtain the parameters under test, including the strain and temperature, which can significantly change $\lambda_{B}$. When a grating is subjected to external strain, the stretching of the grating causes a change in Λ, while the photoelastic effect causes a change in the $n_{eff}$ of the grating. When a grating is subjected to external temperature, its Λ changes due to the thermal expansion of the material, and $n_{eff}$ also changes with temperature [38]. The above changes will eventually lead to a shift in $\lambda_{B}$, which is related to strain and temperature as follows [39]:

(4) $$\Delta \lambda_{B}=\lambda_{B}\left(1-p_{e}\right)\Delta \varepsilon +\lambda_{B}\left(\alpha +\xi \right)\Delta T$$

where $\Delta \lambda_{B}$ is the variation in the peak Bragg wavelength caused by temperature and strain, Δε is the axial strain, ΔT is the temperature difference, and α and ξ are the thermal expansion coefficient and the thermo-optical coefficient of the fiber, respectively. $p_{e}$ is the elastic-optical coefficient [40], which can be expressed as follows [41]:

(5) $$p_{e}=\left(\frac{n_{eff}{}^{2}}{2}\right)\left[p_{12}-\nu \left(p_{11}+p_{12}\right)\right]$$

where ν is the Poisson’s ratio of the material, and $p_{ij}$ is the elasticity coefficient of the fiber.

#### 2.2.3. Scattering in Optical Fibers

**Figure 6:**
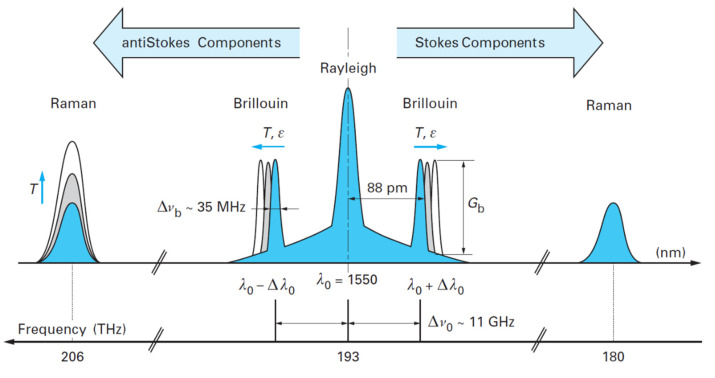
Spectral distribution of Rayleigh, Raman and Brillouin scattering (excitation at 1.55 µm) [43].

When the laser pulse is transmitted through the fiber, light scattering is caused by inhomogeneities in the fiber material. The inhomogeneities include fluctuations in material density, impurities or defects introduced during the manufacturing process, etc. Scattered light can be divided into two types: forward scattering and backward scattering. Backward-scattering-type distributed fiber optic sensors are often used in SHM. Backscattered light consists of Rayleigh, Raman, and Brillouin scattering [42]. Among these, Rayleigh scattered light has the same wavelength as the incident light: see $\lambda_{0}$. In other words, the photon energy is conserved before and after the entire scattering process, hence Rayleigh scattering is also known as elastic scattering. Scattering at wavelengths different from the incident photon wavelength is inelastic scattering. When the wavelength of the scattered light is shorter than the wavelength of the incident light, it becomes anti-Stokes components; when it is on the contrary, it is called Stokes components. The inelastic scattering process can be further divided into Raman scattering and Brillouin scattering. In the case of Raman scattering, the light experiences a wavelength shift of a few tens of nm from the excitation wavelength: see $\Delta \lambda_{0}$ (about 40 nm @ 1 μm in wavelength). In the case of Brillouin scattering, the change in the wavelength of the scattered light is small as compared to that of the incident light, with $\Delta \lambda_{0}$ about 100 pm. The inelastic scattering mechanism leads to generating two symmetric frequencies (two spectral lines) with respect to the laser excitation [43], and their spectral distribution in the case of incident light at a wavelength of 1.55 μm is shown in Figure 6.

When the inhomogeneity scale of the material is short (<λ/10), Rayleigh scattering is produced. From the analysis of electromagnetic field theory, this scattering is a secondary wave emitted by a small particle in the electromagnetic field of light as a forced vibration, forming an electric dipole oscillation. Its main characteristic is as follows:

(6) $$I\left(\theta \right)\propto \frac{1+\cos^{2}\theta }{\lambda^{4}}$$

where $I\left(\theta \right)$ is the intensity of scattered light in the direction of observation at an angle of θ with the direction of the incident light, and λ is the wavelength of the incident light. Since Rayleigh scattering is elastic scattering, the wavelength of scattered light is the same as the wavelength of incident light [44]. It is not sensitive to the temperature around the fiber but can be used to detect the loss distribution of the fiber, such as in crack monitoring [45].

Raman scattering is caused by the interaction of photons propagating in an optical fiber with molecules that are thermally vibrating [46]. More specifically, if a portion of the light energy is converted into the energy of molecular thermal vibrations, then light with a longer wavelength will be emitted. This is called Stokes light, which is almost independent of the temperature [47]. On the contrary, if part of the energy of molecular thermal vibrations is converted into light energy, light with a shorter wavelength will be emitted. This is called anti-Stokes light and is temperature-dependent [48]. Therefore, Raman scattering is widely used for temperature distribution sensing [49]. Raman scattering is a type of inelastic scattered light; it is composed of these two different wavelengths of light, and their frequencies can be expressed, respectively, as follows:

(7) $$\upsilon_{s}=\upsilon_{0}-\Delta \upsilon$$

(8) $$\upsilon_{a}=\upsilon_{0}+\Delta \upsilon$$

where $\upsilon_{s}$ is the frequency of the Stokes light, $\upsilon_{a}$ is the frequency of the anti-Stokes light, $\upsilon_{0}$ is the frequency of the incident light, and Δυ is the molecular vibration frequency of the fiber.

The wavelength shift of Raman-scattered light is determined by the intrinsic properties of the fiber components and the temperature. Thus, the ambient temperature around the fiber can be calculated by testing and collecting the light intensity of Raman-scattered light. The relevant calculation formula is as follows [50]:

(9) $$R_{r}={\left(\frac{\lambda_{s}}{\lambda_{a}}\right)}^{4}e^{-\frac{hc\tilde{\upsilon }}{kT}}={\left(\frac{\upsilon_{a}}{\upsilon_{s}}\right)}^{4}e^{-\frac{hc\tilde{\upsilon }}{kT}}$$

where: $R_{r}$ is the ratio of the peak intensity of anti-Stokes light to that of Stokes; $\lambda_{s}$ and $\lambda_{a}$ are the wavelengths of Stokes light and anti-Stokes light, respectively; h is Planck’s constant, c is the speed of light, $\tilde{\upsilon }$ is their wavenumber separation from the pump wavelength, k is Boltzmann’s constant, and T is the thermodynamic temperature. It can be seen that this relationship is only a function of the temperature and the intrinsic properties of the fiber. Therefore, it can be used to accurately measure temperature without considering the effects of other factors [47,51].

Brillouin scattering is produced by the interaction between photons and sound waves in the medium [52]. The Brillouin frequency shift $\upsilon_{B}$ is linked to the acoustic mode phase velocity [53]. Since Brillouin scattering is an inelastic type of scattering, the frequency of the Brillouin-scattered light is shifted compared to the frequency of the incident light. When the fiber material properties are affected by temperature or strain, the frequency shift of Brillouin-backscattered light changes. The extent of the change in frequency is proportional to the change in temperature and strain, which can be expressed as follows [46]:

(10) $$\Delta \upsilon_{B}=C_{T}\Delta Τ+C_{\varepsilon }\Delta \varepsilon$$

where: $\Delta \upsilon_{B}$ is the variation in the Brillouin frequency shift; $C_{T}$ and $C_{\varepsilon }$ are the temperature coefficient and strain coefficient for the variation in Brillouin frequency shift, respectively [34]; ΔΤ is the amount of change in temperature; and Δε is the amount of change in strain. Therefore, the Brillouin-scattering-based FOS are used for distributed strain or temperature measurements [54].

#### 2.2.4. Optical Time-Domain Reflection

**Figure 7:**
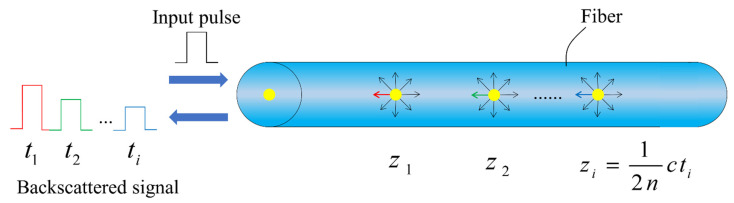
Schematic diagram of OTDR.

Optical Time-Domain Reflection (OTDR) is the most important technique and principle associated with DFOS measurements [23]. It obtains the physical quantities to be measured by detecting changes in the characteristic parameters of backscattered light (light intensity, frequency, polarization state, etc.) [55,56,57]. It determines the spatial location of the physical quantities to be measured by measuring the time of arrival of the backscattered light at the detector [58]. Thus, it is possible to obtain the spatial distribution of the physical quantities to be measured. Its schematic diagram is shown in Figure 7, and the spatial resolution of OTDR is the minimum distance between two scattering positions that can be resolved. If c denotes the velocity of light, n denotes the refractive index of the fiber, and τ denotes the pulse width, the spatial resolution $\Delta Z_{\min}$ can be given as follows [23]:

(11) $$\Delta Z_{\min}=\frac{c\tau }{2n}$$

It can be seen that the spatial resolution of OTDR is determined by the pulse width; for example, if the pulse width is 10 ns, the spatial resolution is 1 m. In general, to obtain a high signal-to-noise ratio due to the weakness of the scattered light signal, it is often necessary to use the average of multiple detection pulses [59]. Moreover, considering each application’s requirements, the optimization of the spatial resolution and the sensing range should be carefully performed [60].

#### 2.2.5. The Sagnac Effect

**Figure 8:**
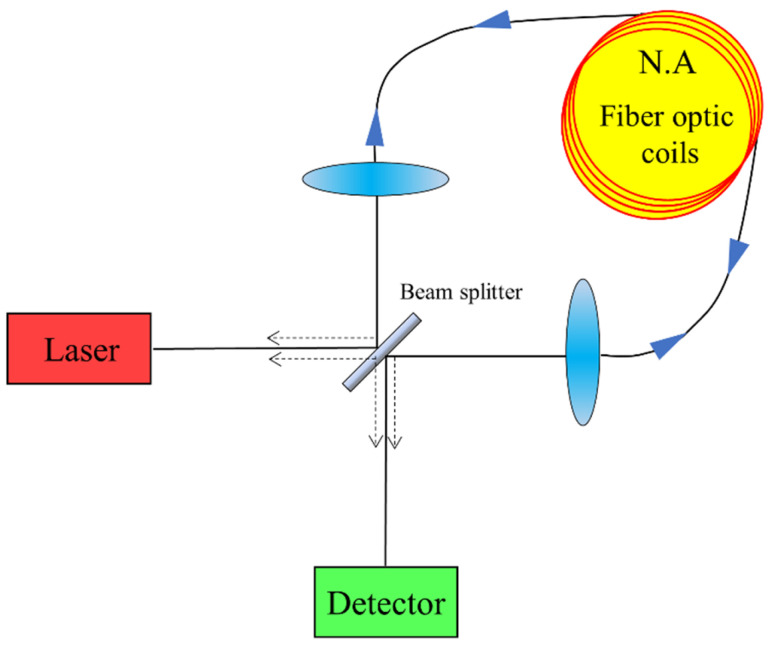
Schematic diagram of fiber optic gyroscope.

Sagnac-effect-based fiber optic gyroscope sensors are mainly used to measure rotation [61]. The simplest fiber optic gyro structure works as shown in Figure 8. The laser is split into two beams by a beam splitter, coupled to the two ends of the fiber. The propagation directions of the beams in the fiber are marked with solid arrows in the figure. After passing through the fiber coil, it is recombined by the beam splitter and shot into the light detector. These two beams will interfere and produce interference fringes when they meet. The propagation directions of the interference beams are marked with dashed arrows in the figure. When the fiber optic gyro is stationary, the interference fringe does not move. On the contrary, when the fiber optic gyro is rotating, due to the Sagnac effect, the two beams of light will produce a phase difference and the interference fringes will move [62]. If Δφ denotes the Sagnac phase shift generated by the fiber optic gyroscope rotation, it can be given by the following equation [60]:

(12) $$\Delta \varphi =\frac{8\pi AN\Omega }{c\lambda_{0}}$$

where A is the area of the fiber coil, N is the number of turns of the coil, Ω is the angular velocity of fiber rotation, c is the speed of light, and $\lambda_{0}$ is the wavelength in a vacuum [63]. Therefore, the rotation angular velocity (Ω) of the fiber optic gyro can be measured by detecting Δφ.

## 3. Progress of Fiber Optic Sensing Technology

### 3.1. Single-Point Fiber Optic Sensors

**Figure 9:**
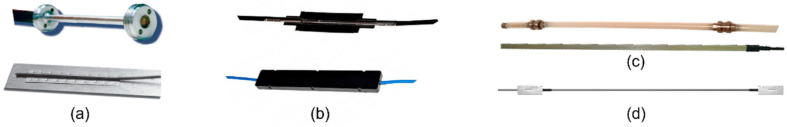
Examples of single-point fiber optic sensors: (**a**) short-gauge EFPI sensors, (**b**) short-gauge FBG sensors, (**c**) long-gauge SOFO sensors, and (**d**) long-gauge intensity-based sensor (photos (**a**–**c**) courtesy of Roctest, Saint-Lambert, QC, Canada, www.roctest.com (accessed on 12 April 2023) and SMARTEC SA, Manno, Switzerland, www.smartec.ch (accessed on 12 April 2023); (**d**) courtesy of OSMOS Group SA, Paris, France, https://www.osmos-group.com (accessed on 12 April 2023); sensor sizes in the figure are not to scale [10].

Single-point fiber optic sensors operate with a single unit to sense and measure changes in parameters in a small area near a predetermined point [34]. Therefore they can offer excellent performance in measuring specific locations. Single-point fiber optic sensors can be divided into two types: short and long gauge. Short-gauge sensors, such as those based on FBG and EFPI, provide the exact value of the measurement, while long-gauge sensors, such as Michelson and Mach–Zehnder Interferometry (also called SOFO sensors), provide the average value of the measurement [10]. Examples of these sensors are shown in Figure 9. In the earlier period, Y. J. Rao et al. [64] proposed an optical fiber FPI sensor based on wavelength multiplexing. It can be used to simultaneously measure the static strain, temperature, and vibration of SHM. This is due to the advantages of FPI sensors, such as the absence of a reference arm, time division, coherent multiplexing, and high resolution [48]. Afterwards, there was an increase in research on system strain and temperature monitoring with fiber optic FPI sensors [65,66,67,68,69]. However, these suffer from disadvantages such as low reusability, difficulty in using them in practical applications, and non-constant sensitivity [70].

In recent years, there has been a great deal of research directed at improving the sensitivity of fiber optic FPI sensors [71,72]. In addition, there are some studies using fiber optic FPI sensors for SHM in high-temperature environments—for example, high-temperature fiber optic FPI temperature sensors [73], high-temperature fiber optic FPI pressure sensors [74], high-temperature fiber optic FPI strain sensors [75], high-temperature fiber optic FPI vibration sensors [76], etc. SOFO sensors are successful low-coherent interferometric sensors for SHM [48]. They have been widely used to monitor the structural health of buildings, dams, bridges, tunnels, pipelines, etc. [77]. They have the advantages of temperature insensitivity, high accuracy, low cost, and good stability [78]; however, SOFO sensor interrogators with high accuracy are costly [79]. Recently, a low-cost SOFO sensor interrogator has been proposed and constructed [79]. It has good measurement accuracy and strong marketability.

**Figure 10:**
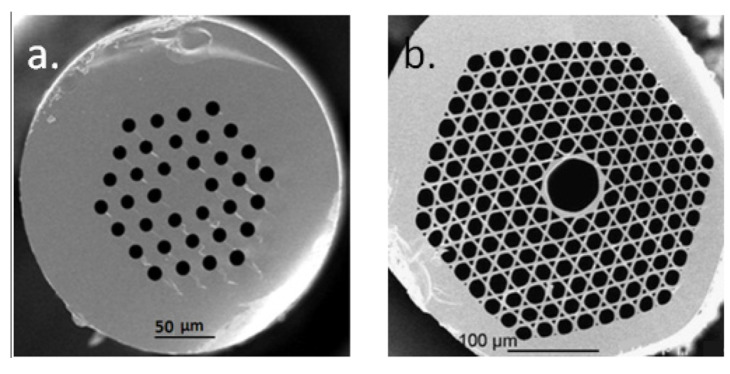
Transverse section of (**a**) MTIR-PCF [81] and (**b**) PBG-PCF [82].

In addition, photonic crystal fiber (PCF) is an emerging single-point sensing type of fiber that has attracted a great deal of interest from researchers in recent years. The most typical feature of a photonic crystal fiber is the presence of many micro vias in its cladding region parallel to the fiber axial direction [80]. Photonic crystal fibers are usually classified into two categories according to the different light conduction mechanisms—namely Modified Total Internal Reflection Photonic Crystal Fiber (MTIR-PCF) and Photonic Band-Gap Photonic Crystal Fiber (PBG-PCF)—and their typical structures are shown in Figure 10 [81,82]. Photonic crystal fiber optic sensors are now capable of being used for SHM. For example, Maintaining Photonic Crystal Fiber (PMPCF) can be used for the SHM of composites [80], and it can obtain accurate strain measurement with very negligible temperature sensitivity. In addition, Chen et al. [83] monitored pressure using a method that measures the birefringence generated in a twin-air-hole PCF. Zheng et al. [84] developed a photonic crystal fiber long-period grating humidity sensor with high sensitivity and selectivity for the non-destructive detection of moisture ingression into structures that can potentially lead to corrosion. Recently, to provide high sensitivity, high birefringence and low confinement loss at the same time, Leon et al. [85] introduced a PCF-based sensor structure for liquid sensing applications.

### 3.2. Quasi-Distributed Fiber Optic Sensors

Quasi-distributed fiber optic sensors contain multiple sensing units to form a sensor array and enable multi-point sensing [86]. They connect single-point fiber optic sensors to form a distribution system through time division multiplexing, frequency division multiplexing, or wavelength division multiplexing techniques [31]. The system combines the characteristics of single-point sensors and fully distributed sensors, so it is called a quasi-distributed fiber optic sensor [34]. This section describes the progress of commonly used quasi-distributed fiber sensors (fiber Bragg grating sensors and long-period fiber grating sensors).

Unlike Fabry–Perot fiber optic sensors, more versatile and flexible solutions are provided by FBG sensors, the gold standards of quasi-distributed sensors [43]. An FBG sensor has the advantages of common optical fiber sensors, but also has unique advantages [87]: the probe size is small, and its diameter is equal to that of the fiber; it allows wavelength modulation, with strong anti-interference ability; it allows the integration of sensing and transmission, with strong multiplexing ability, making it easy to form a sensing network; the measurement object is wide, and it is easy to achieve multi-parameter sensing measurement; etc. Therefore, it has become a compelling research and application hotspot within fiber optic sensors in recent years. FBGs are made by exposing the core of a single-mode fiber to a periodic pattern of intense laser or UV light laterally [88]. The refractive index of the fiber core changes permanently as the exposure pattern changes, and this fixed refractive index modulation is called a grating. However, the FBG diffraction grating disappears at temperatures above 600 °C. To address this problem, Li et al. [89] recently proposed an FBG-based acoustic emission (AE) sensing system that uses a regenerative fiber Bragg grating (RFBG). In addition, femtosecond laser technology has been used for fiber optic microstructure fabrication, including in FBGs [90]. This manufacturing process allows FBG sensors to operate at very high temperatures (even 1300 °C [91]).

There have also been many studies considering how to improve the sensitivity of FBG sensors to temperature or strain [92,93,94,95]; however, the problem of cross-sensitivity between temperature and strain in fiber Bragg gratings is a major obstacle to their practical application [19]. From Equation (4) in Section 2, we can see that the Bragg peak wavelength is sensitive to both temperature and strain. Therefore, it is necessary to distinguish between the two factors—temperature and strain—in the practical application of FBG sensors. A non-exhaustive list of methods that have been proposed for this purpose has been provided previously[24]. Although there is the possibility of using FBG sensors for simultaneous sensing [96], they are primarily used for strain measurements. For strain measurements, the effect of temperature must be compensated for. Several methods for temperature compensation have been provided [97]. However, when the temperature gradient in the structure is not exceeded, the use of a single FBG insulated from the effects of the strain is sufficient to compensate for the temperature effects in the remaining FBGs. In this case, the variations in Bragg wavelength that occur due to strain can be distinguished [41]. In addition, the hybrid fiber optic sensing method is considered a promising approach that can solve this problem [98]. In this approach, two or more FOS operate in combination to eliminate the effects of temperature or strain on individual FOS. For example, FBG sensors and long-period fiber grating (LPFG) sensors are used in combination to differentiate between the effects of strain and temperature in a structure [99].

**Figure 11:**
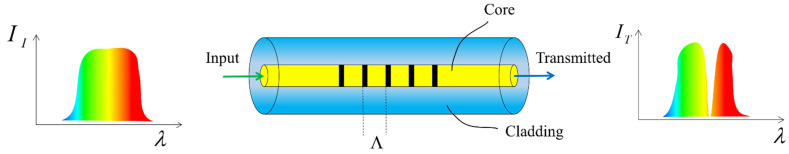
Schematic diagram of the working principle of LPFG.

As another type of quasi-distributed fiber sensor, the LPFG sensor is a new type of fiber-passive device developed in recent years [24]. It forms a periodic or non-periodic refractive index distribution in the fiber core. Due to the coupling effect of internal fields, LPFG transmits light of specific wavelengths with essentially no backscattering, and it is a transmission-type fiber grating [41]. Its sensing principle is shown in Figure 11, where different colors are used to represent different wavelength components in the light beam. The period of long-period fiber gratings is on the submillimeter scale, which makes the fabrication of LPFGs relatively simple. Conventional methods include the amplitude mask template UV exposure method [100], the CO_2_ laser point-by-point writing method [101], and the periodically corrugated template pressing method [102]. The long period of LPFG makes its resonant wavelength and amplitude extremely sensitive to ambient temperature, strain, bending, and torsion [24], and some higher-sensitivity LPEGs have been reported [103,104]. In addition, it plays an increasingly important role in fiber optic sensing because of its high measurement accuracy [105]. Recently, Delgado et al. [106] proposed and discussed a scheme to measure multiple perturbations simultaneously using a single LPFG. Jin et al. [107] proposed a novel sensor structure for the simultaneous measurement of strain and temperature and conducted experimental studies. The structure was created by the weak power modulation of CO_2_ laser exposure on a tapered LPFG. Compared with the transmission spectrum of the tapered LPFG, two peaks appear in the transmission spectrum of the new structure.

In the early 1990s, FBG was introduced to SHM as a very important technology [43]. FBGs are widely used due to their many advantages already described above, such as multiplexing capability and wavelength encoding information, as well as the elimination of power variations [108]. There have been a large number of studies using FBGs for system strain and temperature monitoring [109,110,111,112,113]. New developments in FBG interrogation allow the reading of a large number of tight distributions at high resolution. For example, Sartiano et al. [114] proposed an interrogation technique for cascaded FBG sensors based on microwave photonics under coherent states. This new technique is applicable to position and temperature measurements in cascaded written FBG fibers. FBG sensors have also been explored to monitor circumferential stresses [115] and the inherent frequency of structures [116]. Moreover, they can also be used to monitor corrosion and the resulting cracking in structures [117].

**Figure 12:**
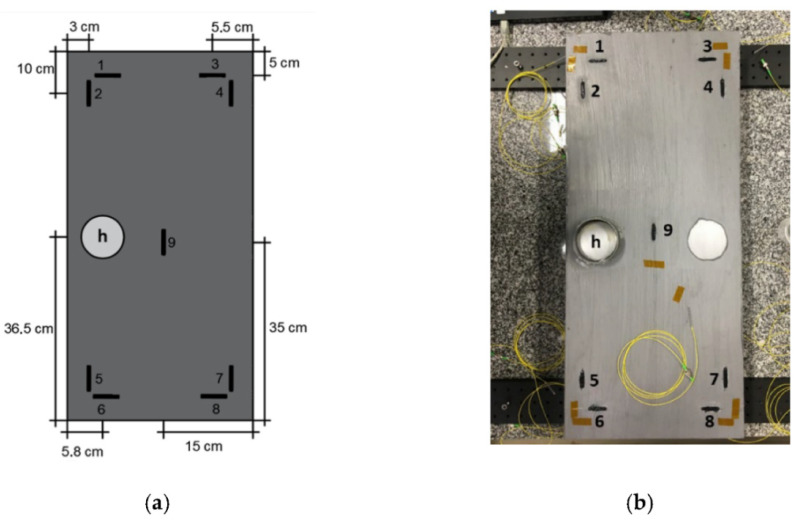
(**a**) FBG sensors’ position and identification and location of the corrosion site on the metal plate; (**b**) photograph of the experimental apparatus [119].

More recently, Mieloszyk et al. presented an FBG-sensor-based SHM system for the monitoring of the technical condition of a 3 kW organic Rankine cycle (ORC) microturbine [118]. Sousa et al. [119] proposed an FBG-based sensing system to detect the initial stages of corrosion and to monitor the thickness loss of a 1020 carbon steel metal plate subjected to controlled corrosion using a NaCl solution. Specifically, they used a 0.1 M NaCl solution to induce corrosion in a region “h” with an area of 40.715 cm^2^ and used nine FBGs. The locations of each sensor and the “h” region are as shown in Figure 12. In addition, Ho et al. [120] presented an SHM system based on FBG sensors which provides accurate time–deformation relations and frequency spectrum results. Moreover, they used the system for the SHM of a linear robot (LR). They used six FBGs to monitor the mechanical vibration and thermal expansion of the LR. The above FBG-sensor-based SHM systems are complex, and to make the operation more convenient, an FBG sensor interrogation consisting of a smartphone and low-cost, off-the-shelf, readily available components has been reported [121].

### 3.3. Distributed Fiber Optic Sensors

DFOS utilize the unique one-dimensional structure and sensitivity of fiber to measure physical parameters along its entire length, treating these parameters as functions of the length of the fiber position. This allows DFOS to continuously monitor the external physical parameters distributed along the fiber path, while also obtaining the state of the spatial distribution of the measured physical parameters and their variation over time. Because they apply to sense measurements that best represent the advantages of fiber optic distribution extension, they have received a great deal of attention and achieved tremendous growth in the past few decades [122]. Their operating principles can be based on reflection techniques (e.g., Rayleigh, Raman, or Brillouin scattering effects [42]) and interferometric techniques.

#### 3.3.1. Reflective DFOS

The most important techniques and principles associated with DFOS measurements are optical time-domain reflectometry (OTDR) and optical frequency-domain reflectometry (OFDR) [23]. Generally, OFDR has a better spatial resolution, while OTDR has a larger sensing range (tens to hundreds of kilometers) [122]. Since the 1980s, OTDR techniques have been applied to Raman-based [49] and Rayleigh-based [123] distributed temperature measurements. A subcategory of Rayleigh sensors is phase-OTDR. Phase-OTDR systems retrieve information about the state of the fiber (due to strain or temperature conditions) by using the amplitude of the retrieved Rayleigh backscatter signal, which changes to reflect changes in the fiber and therefore in the structure being sensed [124]. A more sophisticated linear setup allows the precise measurement of strain and temperature over the entire sensing range, so much so that these systems are among the more successfully commercialized Rayleigh-based DFOS to date [42]. Recently, Filograno et al. [124] proposed a minimally invasive synchronous fiber monitoring system for SHM based on phase-OTDR and evaluated its applicability and performance on a modular Bailey-type bridge at a 1:2.5 scale. Preliminary results of this application of the phase-OTDR system have been reported in Ref [125].

**Figure 13:**
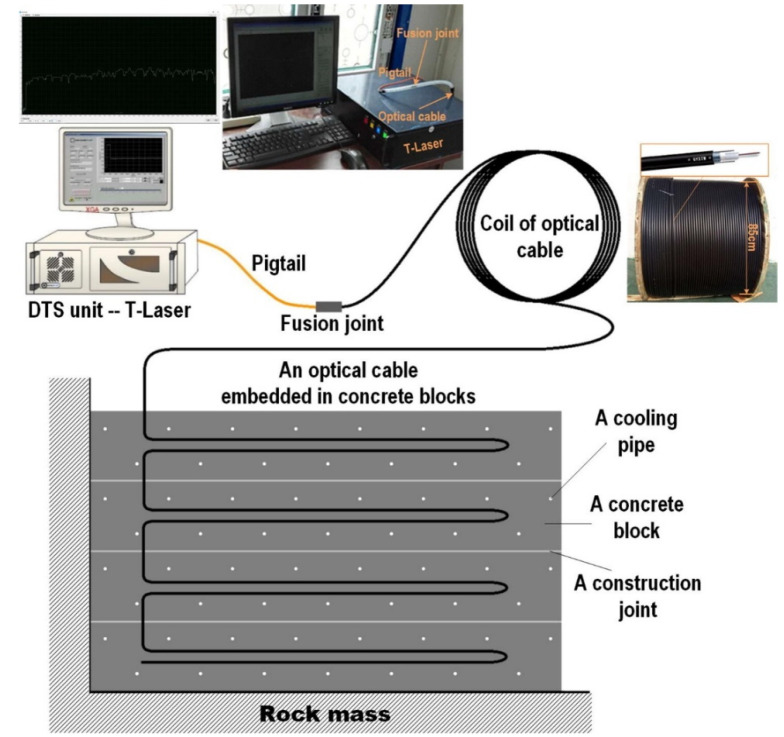
Schematic of the DTS system [128].

As discussed in Section 2, Raman scattering is highly suitable for use in temperature monitoring, and it has been applied in various fields with very successful techniques [126,127]. However, since the Stokes and anti-Stokes light formed by Raman scattering will pass through the same fiber length with different attenuation, an error is introduced if only the reflection ratio, as shown in Section 2, is used to decode the temperature, without considering this attenuation difference. Several methods have been used to solve this problem [44]. In addition, the distributed fiber optic sensor system based on Raman scattering can predict and evaluate the cracking risk of concrete blocks using the measured temperature values as the base data, as has been demonstrated by Ouyang et al. [128]. The distributed temperature sensing (DTS) system they used mainly consists of an optical backscatter reflectometer (OBR) interrogation device and a single fiber optic cable, as shown in Figure 13.

The research on distributed sensing based on Brillouin scattering started late. However, this technology has attracted a number of research efforts recently, because it achieves higher measurement accuracy, measurement range, and spatial resolution in temperature and strain measurements than other sensing methods [129]. The first work on strain sensing based on Brillouin scattering was carried out in 1989 [130]. Subsequently, Tateda et al. [131] described the first distributed demonstration based on excited Brillouin scattering, the so-called Brillouin optical time-domain analysis (BOTDA), which used two back-propagating lasers and exploited Brillouin amplification. Later, Shimizu et al. [132] proposed the Brillouin optical time-domain reflection (BOTDR). It has the advantage of only needing to monitor the system from one end of the sensing fiber. There have been many studies aimed at improving their spatial resolution, such as the Brillouin optical correlation domain analysis (BOCDA) technique [133] and some advanced algorithms [134]. Recently, Noghani et al. [135] proposed a new method called the multiple short pulse BOTDA (MSP-BOTDA), which yielded both sub-centimeter spatial resolution and kilometer dynamic range simultaneously. In addition, OFDR is a viable alternative to overcome the high cost of OTDR for high-spatial-resolution measurements [136]. The use of OFDR for distributed strain measurements was first implemented by Froggatt et al. in 1998 [137]. Subsequently, DOFS based on OFDR and Rayleigh scattering with the millimeter-level spatial resolution was proposed [23]. Its potential to achieve a high spatial resolution in strain measurement has attracted a great deal of attention [31]. In 2015, Stephen T. Kreger’s team developed and demonstrated a novel OFDR and optical phase-based vibration detection and mapping technique [138]. Experimental results show the potential of OFDR-based instrumentation for accurate, high-spatial-resolution, distributed vibration sensing in dynamic environments, which is exactly what is needed for SHM. Recently, Tanimura et al. [139] used an OFDR system with a broad-wavelength fast-scan light source for long-distance strain measurements, with good results. Moreover, Ciminello et al. [140] applied an OFDR system to achieve a spatial resolution of 2.6 mm and a maximum sampling rate of 250 Hz on a 2-m-long fiber. A comparison of different reflective distributed fiber optic sensing techniques is shown in Table 1.

#### 3.3.2. Interferometric DFOS

As a possible low-cost alternative to FBG sensors, there has been considerable progress in the research on polymer optical fiber (POF) sensors with new developments in polymer technology and applications [41]. As a type of interferometric DFOS, POF sensors offer better structural flexibility and resistance to external disturbances and deformations in harsh construction environments. This makes POF sensors suitable for embedded sensors in reinforced structures [146]. Preliminary results show that POF sensors can be used for SHM [118]. Recently, Dong Luo et al. [146] proposed a corrosion-monitoring device based on a tapered polymer optical fiber sensor (TPFS) for steel bars. This is a novel non-destructive approach to monitoring the corrosion condition of steel reinforcements. In addition, an increasing number of studies are using POF sensors for the SHM of bonded joints [147]. To obtain better performance, a microstructure design of POF is required, forming microstructured POF (mPOF) [24]. It is considered to be the counterpart version of photonic crystal fiber on a polymer fiber [41]. There have been several studies using mPOF for SHM. For example, Garcí et al. [41] described the use of long-period gratings (LPG) in mPOF, rather than FBG, as an option for measuring strain in aircraft structures.

In addition to POF sensors, fiber optic gyroscope sensors (FOGs) are considered to be the most successful and sophisticated interferometric DFOS in fiber optic sensing technology [148]. They are superior angular velocity sensors that have been widely used in many important applications [149]. Compared with mechanical gyroscopes, they have high sensitivity, a low cost, no moving elements, and good robustness [63]. In recent years, resonant fiber optic gyroscopic sensors (RFOGs) have become a hotspot for FOG research. Wang et al. [150] have published a review summarizing the development of RFOGs in recent years and analyzing the prospects and research challenges of RFOGs. In addition, Advanced Navigation recently announced a new fiber optic gyroscope (FOG)-based inertial navigation system (INS), the Boreas D70, designed to open up new application possibilities for fiber optic gyroscope inertial navigation such as self-driving cars and aircraft.

### 3.4. Fiber Optic Sensing Algorithms

In addition to advances in fiber optic sensors, advances in algorithms have led to the emergence of fiber optic sensing technology in SHM applications. Even in the last 20 years, the development of SHM technology has been more related to algorithmic improvements [151]. For example: when measuring some parameters of a structure (such as temperature or strain) with fiber optic sensors, the admixture of noise is unavoidable. Data denoising is crucial to the implementation of FOS technology, and the corresponding denoising algorithms play an indelible role in this process. Common denoising methods that have been proposed include wavelet transform [152], adaptive filtering techniques [153], image and video denoising algorithms [154,155], and deep learning algorithms such as convolutional neural network denoising [156]. Again, some of the previously described problems of fiber optic sensors cross-sensitive to temperature and strain have been solved by some machine learning algorithms [157,158,159]. In recent years, many algorithmic advances have improved the data processing speed and measurement accuracy of fiber optic sensing systems. For example, Zhang et al. [160] proposed a new algorithm for the BOTDR sensing system based on a radial basis function neural network (RBFNN). The algorithm not only improves the data processing speed but also ensures the accuracy of the measurement. Wu et al. [161] demonstrated a BOTDA sensing system using a support vector machine (SVM) for high-speed temperature measurements. This increases the speed of data processing even further, with the algorithm achieving a 100-fold increase in data processing speed compared to traditional data processing techniques. Silva et al. [162] demonstrated ML-based data processing using a commercially available Brillouin-based distributed temperature sensing system (AP Sensing, N4385B). In summary, as can be seen from the above examples, improvements in data processing and computational algorithms have provided greater convenience for data analysis with fiber optic sensing technology. For more detailed related applications, interested readers can see the reference [163]. Accordingly, the discussion of the algorithm is not the focus of this paper, and so interested readers can go through the latest reference [78,164] to learn more about it.

## 4. Applications of Fiber Optic Sensing Technology

### 4.1. Bridge Structure Health Monitoring

Fiber optic sensors have proven to be the best choice for the long-term health monitoring of concrete bridges due to several advantages, as described in Section 2 above [63]. Previously, FBG sensors have been successfully used to monitor the dynamic response of bridges [165] and the vibration characteristics of cables on bridges [166]. With the development of distributed fiber optic sensors (DFOS) in recent years, several researchers have verified the feasibility of monitoring fiber-reinforced polymer (FRP) composite bridges with a Rayleigh-scattering-based DFOS technique based on experimental and numerical results [167]. Some other researchers have used the Brillouin optical time-domain reflectometer (BOTDR) technique to monitor the Nine Wells Bridge, and the data obtained were discussed and analyzed. Other researchers have developed a new type of DFOS, which is based on the optical backscattered reflection (OBR) technique [168]. The capability and good performance of this DFOS for the long-term monitoring of bridge structures have been confirmed by some fatigue tests [169]. Of course, DFOS can also be useful for bridge deflection monitoring [170], and the latest advancement here has been the use of polymer optical fiber sensing (POFS)-based technology to monitor bridge deflection [171]. Since DFOS is less commonly used in practice on structures outside the laboratory, Novák et al. [172] successfully demonstrated the potential of using DFOS to detect and monitor traffic-induced crack development in field experiments on bridges with traffic vibrations. Table 2 shows the main bridges in the world equipped with SHM systems with fiber optic sensors.

### 4.2. Tunnel Structure Health Monitoring

In recent years, DFOS has become increasingly popular for tunnel monitoring. In 2017, during the construction period of a highway tunnel in Slovakia, Marcel Fajkuset et al. [180] used BOTDR-based DFOS to monitor the structural loads in it. As SHM in shield tunnels is not mature enough, Wang Tao et al. [181] proposed a DFOS-based SHM system for operating shield tunnels, and this was experimentally validated on the Nanjing Yangtze River Tunnel over a period of 55 days and obtained good results. Afterward, Gómez et al. [182] presented the application of DFOS to the SHM of the TMB L-9 metro tunnel in Barcelona and studied the reliability of the installed DFOS. In addition, Monsberger et al. [183] designed a DFOS capable of application in shotcrete tunnel cross-sections as well as in shaft linings. It combined DFOS strain measurements and geodetic displacement readings, and the system achieved a high spatial resolution in the centimeter range.

### 4.3. Railway Structure Health Monitoring

**Figure 14:**
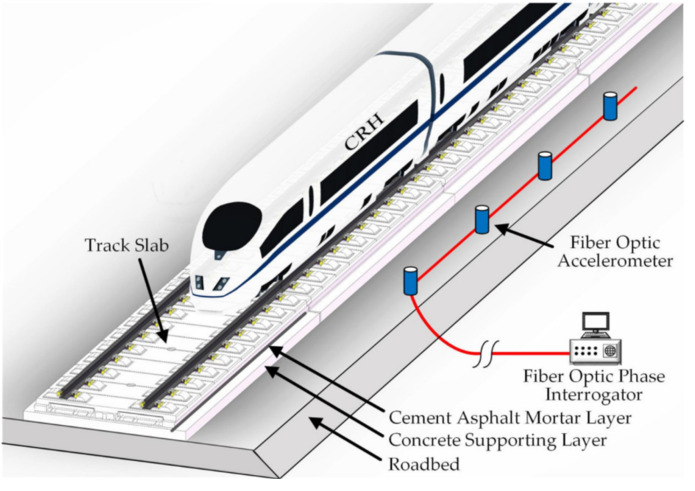
Composition of the experimental system [188].

Railway infrastructure and systems play an important role as an efficient mode of transportation [87]. In recent years, a large number of FOS have been used for SHM on railways. Similarly, FBG sensors have been used to measure strains in railroad concrete rail sleepers [184]. Recent developments are mainly focused on the applications of DFOS for SHM in railways. For example, Rayleigh-backscatter-based DFOS has been used to monitor the dynamic strain of railroads under different vibration conditions [185]. In addition to being able to monitor strains, DFOS can also be used to monitor the transverse buckling of rails under axial loads with different boundary conditions [186]. In addition to DFOS, a hybrid fiber optic sensor (HFOS) system has been used to monitor track geometry in real-time [187]. It is based on FBG and Raman-distributed temperature sensing (RDTS). Its false-alarm-free capability was verified in preliminary field tests. There are also fiber optic acceleration sensors that have not been previously described in this paper. This technology is also capable of being used to record train vibrations and was tested in the field for over a year on the Beijing–Shanghai high-speed railroad by Zhang et al. [188]. Figure 14 illustrates the configuration of the long-term real-time train-induced vibration signal acquisition system they used.

### 4.4. SHM in Other Fields

Of course, in addition to the above applications, other fields also demonstrate applications of fiber optic sensing technology for SHM, such as aerospace [189], composites [190], geotechnical engineering [191], the power industry [192], road infrastructure [193], the petrochemical industry [194], agriculture [195], buildings [196], etc. The references therein are typical review articles or recent studies of FOS applications in the corresponding fields. This is to facilitate a detailed understanding for readers interested in specific areas. In conclusion, fiber optic sensors offer new possibilities for SHM in various fields. However, many studies are still needed to continuously apply some new fiber optic sensing technologies in some practical environments with more complex conditions.

## 5. Vision Sensing Technology

### 5.1. Basics and Principles of Vision Sensing Technology

**Figure 15:**
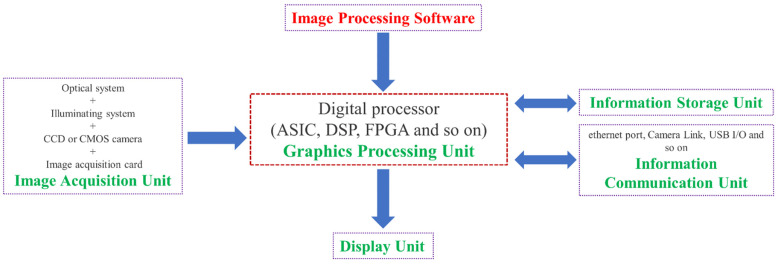
The components of the vision sensing system and the relationship between them.

Computer vision, a simulation of biological vision using computers and related devices, is an important part of the field of artificial intelligence (AI) [197]. The goal of its research is to provide computers with the ability to perceive three-dimensional (3D) environmental information through two-dimensional (2D) images [198]. In recent years, with the development of digital camera technology and computer processing ability, various applications of computer vision technology have become possible [199]. One of the applications is the use of a vision sensor system for SHM [25]. The vision sensor system is an SHM system for the long-range, non-contact, non-damage monitoring of structures [197]. It is composed of cameras and vision sensors as the main components, with computer image recognition and tracking algorithms as the core. The components of the vision sensing system and the relationship between them are shown in Figure 15. A vision sensor is an instrument that uses optical elements and imaging devices to obtain image information about the external environment. The vision sensor is the direct source of information for the entire vision sensor system. It consists mainly of one or two image sensors, sometimes with light projectors and other auxiliary equipment. The primary function of the vision sensor is to acquire enough of the raw images to be processed by the system. Image sensors can use laser scanners, Charge Coupled Devices (CCD), or Complementary Metal-Oxide-Semiconductors (CMOS), etc.

The main principle of vision sensor technology is image processing, where the image captured by the camera is processed to calculate the characteristic quantities of the object (area, the center of gravity, length, position, etc.) and output the data and judgment results [200]. Vision sensors can capture light from an entire image with thousands of pixels. The clarity and detail of an image are usually described in terms of resolution, expressed in terms of the number of pixels. After capturing an image, the vision sensor compares it to a baseline image stored in memory to perform an analysis. This can be used to directly identify fractures or spalling [201,202].

**Figure 16:**
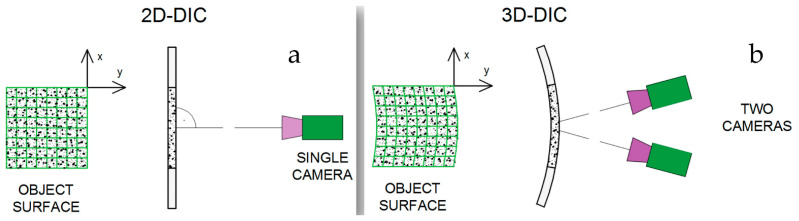
(**a**) 2D DIC system; (**b**) 3D DIC system (modified from [208]).

As an important component of visual sensing technology, digital image correlation (DIC) has been widely used in structural health monitoring (SHM) [203]. DIC is an optical measurement method that can obtain information about the deformation field (such as displacement field, strain field, etc.) on the surface of an object. It needs to perform certain correlation operations on the two speckle patterns of the object surface taken before and after the deformation. It was first proposed by Yamaguchi et al. in 1981 [204]. These researchers measured the in-plane displacement of the object by creating stripes on the surface of the object and using a laser speckle in combination with a linear sensor. However, the accuracy of the test results was low because of the non-linear experimental process. Later, Peters and Ranson [205] proposed to divide the deformation field into different size analysis regions (i.e., subsets), and performed correlation calculations on the subsets before and after the deformation. This was also early two-dimensional digital image correlation technology (2D DIC). However, 2D DIC can only measure the in-plane displacement of the object, and cannot accurately calculate the off-plane displacement. To overcome this limitation, Luo, Chao, et al. [206] combined the concept of binocular stereo vision with the 2D DIC technique and proposed a three-dimensional digital image correlation measurement method (3D DIC). It captures information about the object by using two cameras at the same time to build a three-dimensional model of the object, and can analyze the three-dimensional image of the object. The compositions of the 2D DIC system and 3D DIC system are shown in Figure 16. As another new 3D expansion of 2D DIC technology, Digital Volume Correlation (DVC) is a high-precision measurement by comparing the results of volumetric photographs obtained with 3D photographic devices [207].

### 5.2. Progress of Vision Sensing Technology

Speckle patterns are an important feature of the DIC technique, and their quality has a significant impact on measurement accuracy. Therefore, the progress of DIC technology is inseparable from the optimization of speckle patterns [209,210]. In addition, some of the advances in DIC technology are focused on the number of cameras. For example, with the addition of additional optical elements, 3D deformation measurement of structures can be achieved with a single camera [211]. This technique negates the need for two precisely synchronized cameras and significantly minimizes hardware costs. Other studies have expanded the measurement range and improved measurement accuracy by increasing the number of cameras [212,213]. In addition, camera calibration is critical in the implementation of DIC technology, and there is a well-established and widely accepted standardized technique [214]. Of course, new calibration methods are constantly proposed to accommodate more complex application scenarios [215,216].

In addition to advances in analysis methods and operations, advances in software algorithms have contributed to making DIC technology a robust and mature technology. There have been many reviews summarizing the progress of DIC algorithms, and the next section, in this paper, focuses on summarizing the many vision-based algorithms proposed in recent years. It includes edge detectors [217], thresholding [218], segmentation [219], and filter-based algorithms [220]. Especially when using modern algorithms that implement higher-order interpolation filters, the bias caused by the image-matching algorithm is very low, even for very poor speckle quality [221]. In addition, Yang et al. [222] applied the discrete cosine transform and wavelet transform techniques to the DIC algorithm to obtain accurate displacement and strain fields by using only 5% of the original image size. With the advancement of computer computing power, in addition to DIC-related algorithms based on global finite elements [223], methods based on machine learning and artificial intelligence have emerged [224,225]. Among the emerging machine learning algorithms for signal processing, convolutional neural networks (CNN) for local damage assessment and anomaly detection of structures have become a notable trend [226]. However, many machine learning algorithms work well only under a common assumption that the training and test data have the same distribution and feature space [227]. This assumption is obviously difficult to satisfied considering the practical applications, especially in the execution of SHM. To solve this problem, transfer learning (TL) has been taken as a promising approach [228]. Of course, the latest development of the digital twin (DT) offers another new option for better implementation of SHM [229]. In conclusion, the continuous improvement and development of the DIC algorithm makes DIC matching faster and more accurate, while the integration with some popular algorithms makes it more advanced and intelligent.

### 5.3. SHM Applications of Vision Sensing Technology

Thus far, the vision sensor system has been studied and applied in both laboratory and practical engineering, and reference [25] has given a good overview of its development and application. This paper focuses on some recent advances in laboratory investigations and field applications of vision sensing technology for SHM in recent years.

#### 5.3.1. Laboratory Investigations

**Figure 17:**
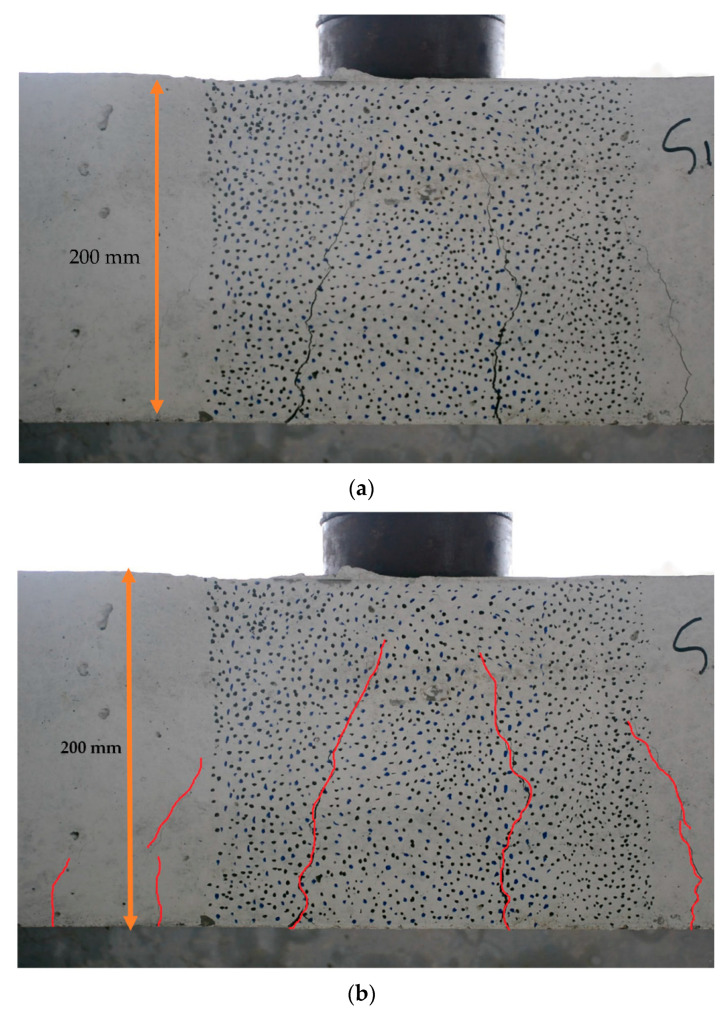

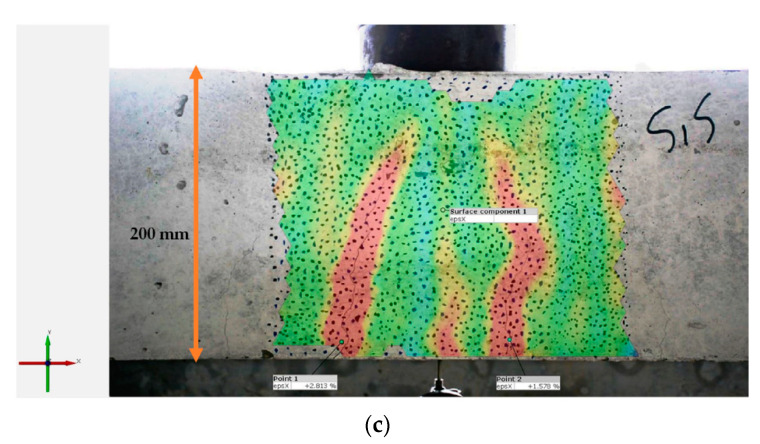
(**a**) Reinforced concrete beams to be checked for cracks; (**b**) Cracks identified with a marker pen; (**c**) Cracks within the speckle zone identified with DIC (modified from [203]).

The development of various technologies continues to drive the advancement of DIC technology. Experiments related to the use of DIC technology for measuring strain and displacement of structures have been increasing and have matured. For instance, the 2D DIC method was used in the laboratory by Hoult et al. [230] to measure strains in steel specimens under uniaxial loading; the results were compared with those of strain gauges and the two were in high agreement. In the experiments of Ellenberg et al. [231], the 3D DIC method was used to measure the static displacements of specimens in the laboratory with sub-pixel accuracy. In addition to measuring strain and displacement of specimens, vision sensing techniques are more often used to probe crack development in the laboratory. One of the most typical tests is the measurement of crack width and crack slip in reinforced concrete (RC) structures using the DIC technique [232]. Figure 17 shows an example of crack monitoring in the laboratory using the DIC technique. The study of reinforced concrete elements is also aimed at their shear mechanism; a thorough review of related studies has been presented in reference [201].

**Figure 18:**
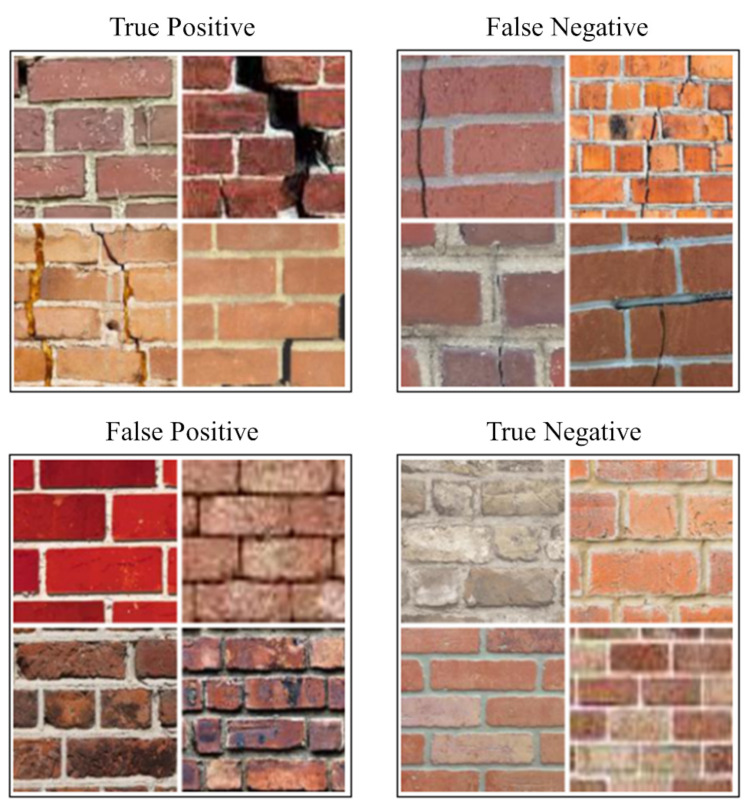
Examples of images used in crack detection [201].

In addition to RC structures, Hallee et al. [201] tested their ability to capture cracks in masonry structures by training CNN. The results show that the CNN architecture suitable for concrete crack detection is not applicable to the detection of cracks in masonry structures. The images used in CNN-based crack detection are illustrated in Figure 18. The latest experimental investigation of using DIC technology for SHM is mainly to investigate the reliability of new sensing technologies, such as fiber optic sensing technology. There are also studies to demonstrate the advantages and development potential of combining DIC and other sensing technologies. The integration of fiber optic sensing technology and DIC technology mentioned in this paper will be described in detail in Section 5.4.

In addition to the normal test environment, DIC technology has attracted many scholars to use it in the field of high-temperature measurements and to conduct in-depth research. Since Turner and Russell first discussed the limitations and development potential of the DIC method for high-temperature applications in 1990 [233], high-temperature DIC research with increasing upper-temperature limits has been conducted. In recent years, Liang et al. [234] used in situ scanning electron microscopy (SEM) to measure the high-temperature deformation and fatigue properties of nickel-based single crystal high-temperature alloys up to 800 °C. In 2020, Pan et al. [235] applied DIC to the ultra-high temperature field (over 3000 °C) using an electron beam heating technique and successfully performed full-field thermal strain measurements on tungsten specimens. In the same year, Chen et al. [236] used a single polarization camera and a polarizing beam splitter to synchronously capture two full-frame sub-images with orthogonal polarization directions. They built a single-camera stereo polarization DIC system and validated the effectiveness and accuracy of the proposed technique through high-temperature testing. Similarly, Wang et al. [237] proposed a full-field thermal deformation measurement method based on high-temperature DIC using a polarization camera as the image acquisition system in combination with the optical filtering system. Finally, they used the built system to perform thermal deformation tests on ceramic materials. The results show that the system can achieve high contrast and clear image acquisition under high-temperature conditions. The fundamentals of high-temperature DIC and some of the challenges it faces are described in detail in a recent review; interested readers can read the reference [238] for further information.

#### 5.3.2. Field Applications

**Figure 19:**
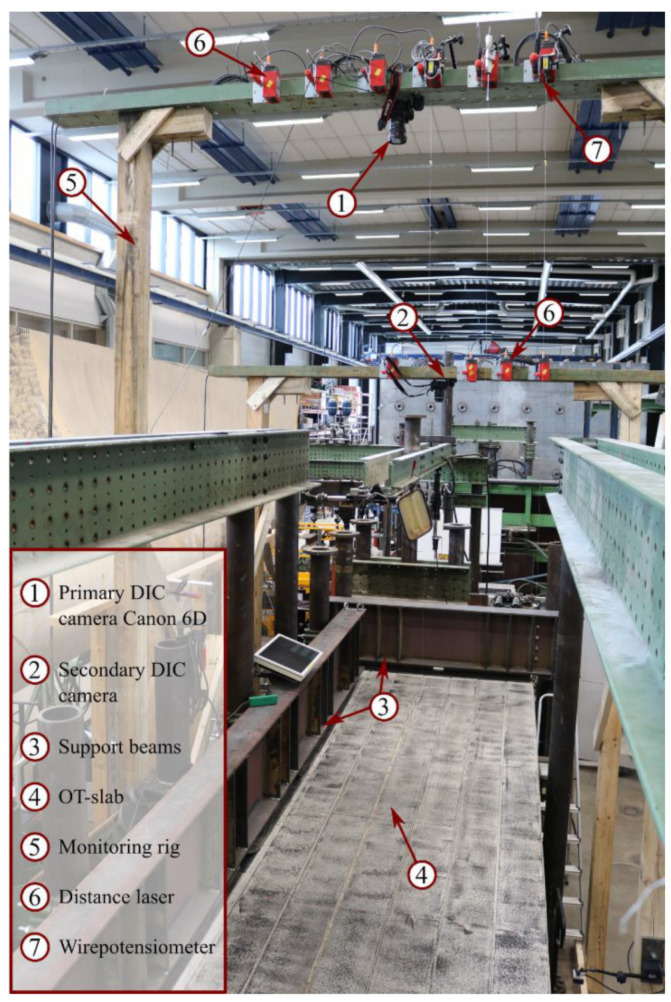
Test setup with monitoring rig separate from test setup [245].

With the development of DIC technology and its wide application in the laboratory, its advantages are becoming more and more obvious. It not only has the advantages of non-contact, simple operation, full-field measurement, a wide range of use, and high measurement accuracy, but also has very low requirements for the device [239]. Therefore, DIC technology has been widely used in engineering projects to monitor the health of structures. McCormick and Lord used the 2D DIC method to measure the vertical displacements of a highway bridge deck with four 32-ton trucks loaded statically [240]. Yoneyama et al. [241] used the 2D DIC method to estimate the deflection of the main beam of a bridge carrying a 20-ton truck. Aiming also to measure bridge deflection, Winkler et al. [242] monitored and evaluated the Warton bridge with a three-car train operating on it using DIC. Recently, to obtain full-field deflection measurements of bridges more easily, Tian et al. [243] proposed a new method based on off-axis DIC, which uses a “straight-line fitting scheme” to measure the full-field deflection over multiple points. This measurement is consistent with the data obtained with conventional displacement meters. Among the reported factors affecting measurement accuracy are internal factors (e.g., relevant algorithms, mathematical functions, etc.) and external factors (e.g., measurement environment, instrument parameters, and target characteristics). The effectiveness and practicality of the method have been verified by the application of SHM for indoor cantilever girders and high-speed railway concrete bridges. In these field test operations, the DIC is implemented by mounting the target to the bottom of the girders to measure the bridge deflections [244]. In addition to measuring bridge deflection, DIC is also used to monitor the development of cracks in reinforced concrete bridges in the field [245]. The field test setup used in the study by Christensen et al. is shown in Figure 19. The monitoring devices used include Linear Variable Differential Transformers (LVDTs), wire potentiometers and distance lasers for deflection measurements, strain gauges for transverse reinforcement strain monitoring, and 2D DIC for full-field surface assessment of deformation and cracks.

The above-mentioned field applications of DIC technology are generally used for static monitoring of structures. Some researchers have also investigated the use of the DIC technique for the dynamic monitoring of structures accordingly. For example, the combination of DIC technology and UAV technology has been used for the health inspection of structures. For example, Reagan applied the 3D DIC method to the long-term monitoring of bridge deformation using an unmanned aerial vehicle (UAV) carrying a stereo camera [246]. Narazaki et al. [247] proposed a vision-based autonomous UAV navigation planning method for rapid post-earthquake inspections of reinforced concrete railway bridge viaducts. The results show that the method can achieve centimeter-level accuracy and has great potential for application in post-earthquake structural inspection. Du et al. [248] investigated the dynamic response of the stay cable Guanhe bridge by using two measurement techniques, DIC and digital image processing (DIP), in combination with a conventional accelerometer.

**Figure 20:**
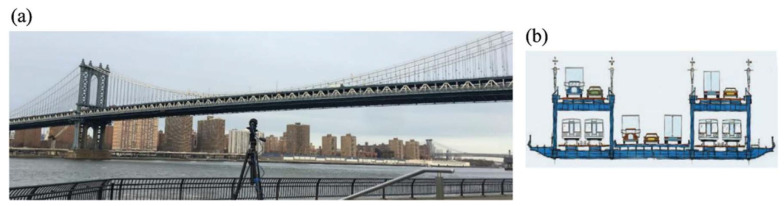
Field measurement of Manhattan Bridge: (**a**) test setup and (**b**) cross-section [249].

Measuring the dynamic response of a structure under excitation, such as displacement and acceleration, and performing modal analysis and system identification to obtain the dynamic characteristics of the structural system is an important element of SHM. Several other vision-based methods have been used for the dynamic monitoring of structures. Feng et al. [249] first demonstrated the accuracy of vision sensors in dense full-field displacement measurements by experimenting with a simply supported beam structure in the laboratory. In this experiment, the structure frequency and vibration patterns were obtained from the displacement measurements of 30 measurement points of the structure by one camera. They were in high agreement with the results obtained using six accelerometers. The vision sensor was subsequently applied to remote (camera at approximately 300 m from the bridge’s mid-span location), real-time, and multi-point (3 measurement loci) dynamic displacement measurements of the Manhattan Bridge under the excitation of an operating train, as shown in Figure 20.

With the trend of digitalization, Min et al. [250] explored the feasibility of real-time dynamic displacement monitoring with smartphones to make computer vision techniques simpler and easier to implement. They developed a new application for the iPhone in the iOS environment to perform vision-based displacement measurements. In an outdoor shaker test, the method achieved sub-millimeter displacement measurement accuracy at a distance of 33 m from the target, and the dynamic displacement measurement results matched well with those of a conventional laser displacement sensor. Subsequently, Kromanis et al. [251] demonstrated the capability of smartphone technology to provide accurate information about structural deformation when combined with appropriate image processing software in a laboratory setting. Interested readers are referred to the recent comprehensive review on smartphone-related sensor technologies and their applications in SHM by Malekloo et al. [252]. In conclusion, vision sensing technology applied to SHM is developing rapidly and it has been considered a new generation of sensing technology in SHM applications [10].

From the above description, it is clear that vision sensing technology has the following main advantages compared to fiber optic sensing technology:

- High accuracy: the vision sensor system can directly measure the overall displacement of the structure; the overall displacement of the structure more directly reflects the changes in the overall stiffness of the structure, and it has the characteristics of artificial intelligence, so it can reflect the overall state of the structure more accurately. In addition, the test instrument can be placed further away—even up to several hundred meters away—and can also achieve the desired accuracy.
- High flexibility: through the lens of the camera and computer image processing algorithms, with the use of non-contact vision sensing technology for displacement measurement, it is not necessary to consider the complex method by which to embed the sensor into the structure; vision sensing technology can be in the form of non-contact distribution sensing technology, tracking multiple changing targets over a long distance to obtain synchronized data.
- Low cost: vision sensing systems do not require a large amount of time for sensor installation, do not require physical contact with the structure, and can be controlled remotely, saving time and equipment costs, especially for bridges. Moreover, no traffic control is required for installation [25].

### 5.4. Integration with Fiber Optic Sensing Technology

The advantages of fiber optic sensing technology and vision sensing technology have been described above in this paper. The main feature of fiber optic sensing technology is contact measurement, while vision sensing technology is non-contact measurement. For SHM, it is a multidisciplinary research project. Both contact and non-contact measurements on structures are necessary to obtain a more accurate health status of them. There have been a large number of experimental studies combining these two technologies, in particular DFOS and DIC. This integration will facilitate the mutual verification and validation of the results measured by both techniques. In addition, it allows the integration of external strains measured by vision sensing techniques with internal strains measured by fiber optic sensing techniques, which allows for more comprehensive health information of the structure and more accurate predictions.

**Figure 21:**
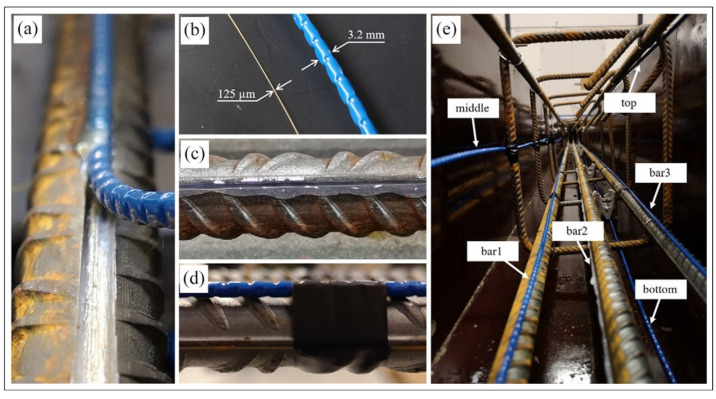
Installation of the optical fiber sensors: (**a**) installation of robust DOFS cable in a reinforcement bar by inserting it into a previously milled groove; (**b**) comparison of thin and robust DOFS; (**c**) installation of thin DOFS on the surface of a reinforcement bar by bonding it with cyanoacrylate adhesive and protecting it with silicone; (**d**) installation of robust DOFS on the surface of a reinforcement bar by mechanically anchoring the cable to the reinforcement with electric tape; and (**e**) multi-layer configuration of embedded DOFS in the beam specimens (modified from [254]).

In 2021, Berrocal et al. [253] combined the DIC technique with fiber optic sensing to investigate the accuracy and reliability of DFOS under monotonic and cyclic loading. They also verified the ability of DFOS to estimate the crack width, where the role of DIC is reflected in obtaining accurate and reliable information about the crack formation and development. The experimental results show that most of the errors in crack location and crack width calculated based on DFOS strain measurements are less than ±3 cm and ±20 µm, respectively, compared to the DIC results. These results are in good agreement with their subsequent studies. In subsequent studies, their main contribution was to demonstrate that robust fiber optic cables with protective cladding, well-suited for field applications, also have good strain measurement performance [254]. They have a certain strain attenuation compared to the commonly used thin polyimide-coated distributed fiber optic sensors bonded to steel bars. The installation of these two fiber optic sensors is shown in Figure 21.

Some studies similarly use DIC results as an accurate and reliable check. Sawicki et al. [255] evaluated the ability of two fiber optic sensors to monitor strains and discontinuities in Ultra-High-Performance Fiber Reinforced Cementitious composites (UHPFRC) by comparing them with the DIC method and the extensometer method. Experimental results show that the use of the extensometer is limited to the elastic and strain-hardening phases for all phases of the structural response of the substrate. The DIC technique is suitable for detecting and tracking localized fictitious cracks during the cracking phase of a structure, but not for observing the strain changes in the structure during the elastic phase. This is due to the large measurement noise that can exist in the elastic phase. DFOS technology enables precise monitoring of the elastic, strain hardening, and softening stages of UHPFRC. Table 3 shows a comparison of the application ranges of the measurement methods they used.

**Figure 22:**
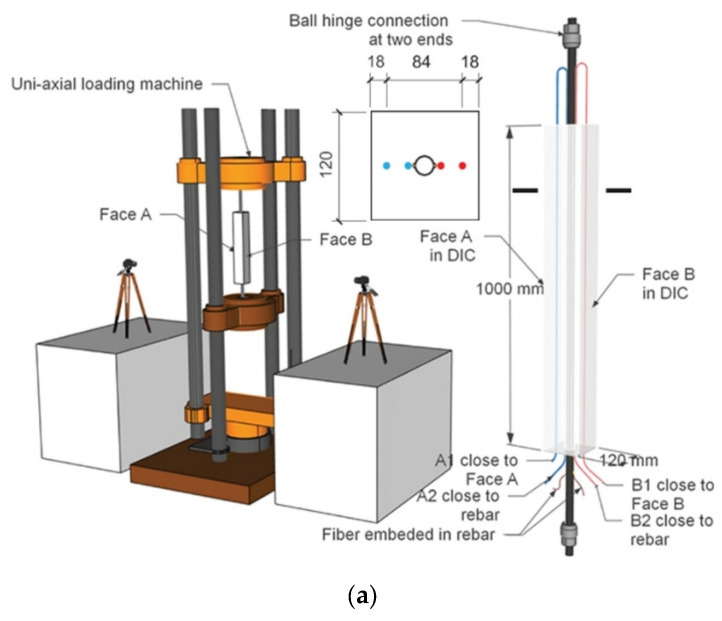

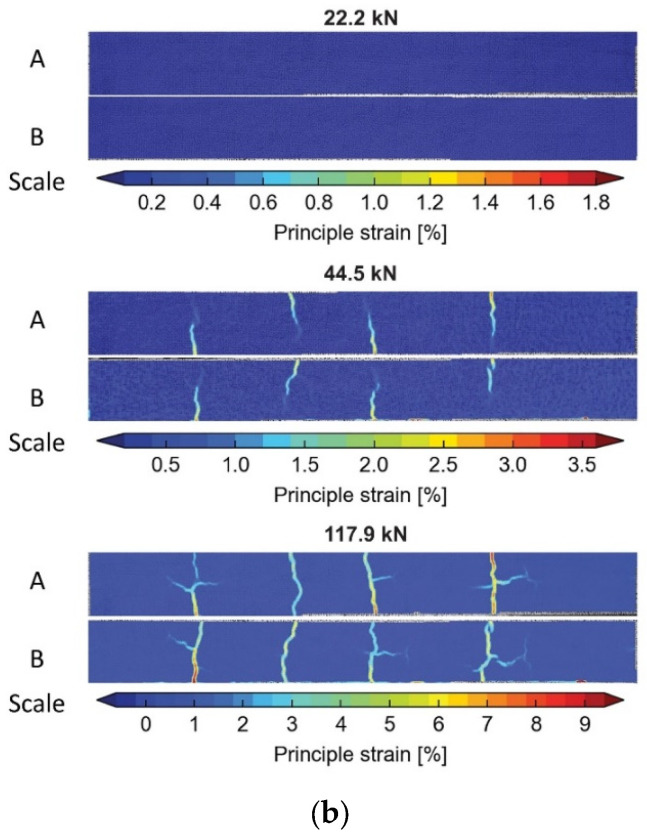
(**a**) Illustration of test setup (sketchup model); (**b**) DIC results of the face A and face B of the specimen at three different loading levels (modified from [256]).

Similar to the previously described study, to obtain improved accuracy and resolution, Zhang et al. [256] applied OFDR to investigate the ability of DFOS to detect concrete cracking and large-strain steel deformation. In the process of their research, they used several types of fiber optic cables. To provide practical guidelines for choosing cables, they established a framework for evaluating cable sensitivity and survivability. Similarly, to check the accuracy of DFOS measurements, they also used DIC results for comparison and analyzed the results of both. The corresponding model of the test setup arrangement and the DIC results of the specimen under different loading levels are shown in Figure 22. To investigate the bond stress and surface slip between concrete and steel rebars in cracked and non-cracked RC tensile members of different sizes, Bado et al. [257] performed extensive experiments by applying DOFS to the surface of the steel rebars and measured these parameters. To verify the accuracy of these measurements, they also introduced DIC technology as a profiling validation and troubleshooting tool. The DIC was used to collect surface data of the RC structure, which was checked against the local strain data of the reinforcement measured by DOFS. Finally, they observed a good agreement between these two results. This demonstrates the viability and integration application potential of the DOFS/DIC combination for RC structural testing.

**Figure 23:**
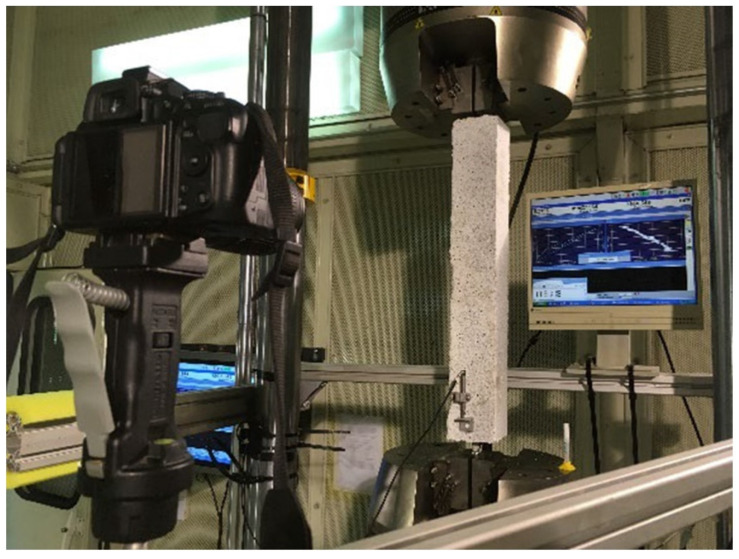
Experimental setup with a photo camera set to take pictures at regular intervals [258].

There have been some explorations of the combined application of DOFS and DIC. For example, Bado et al. [258] investigated the potential and performance of combined DOFS and DIC techniques for internal and external strain monitoring in RC ties. Their results indicate that the combined use of DOFS and DIC provides researchers with an excellent monitoring system. This system provides a complete picture of the internal strain distribution of the member through continuous interaction and inspection between external and internal strain measurements. The experimental setup that the researchers used is shown in Figure 23. The concrete mix they used was formed by mixing water and other materials in a certain ratio, and the FOS were bonded to the steel bars in the setup. The entire structure was deformed by the action of the hydraulic actuator and the camera was set to take pictures at regular intervals. Cantone et al. [259] considered that the DIC technique can only collect concrete surface data and cannot directly measure the local strain state of the reinforcement. They obtained high-quality results by attaching Fiber Optical Measurement systems (FOM) to the surface of the rebar. This takes advantage of the fact that the FOM allows high-strain gradients to be observed in the bar along its axis for different locations in the cross-section. They combined these two techniques to test three different types of structural elements to re-investigate the interaction between reinforcing steel and concrete from a new perspective. Finally, they show the implications of such interactions on a series of phenomena associated with brittle failure modes.

**Figure 24:**
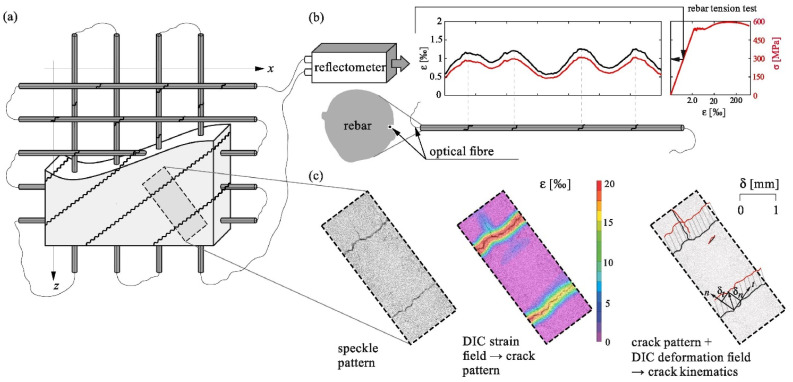
Combined use of DIC and DFOS: (**a**) general setup of possible structural concrete experiment with speckled surface and reinforcing bars instrumented with optical fibers; (**b**) DFOS continuous strain measurement along one bar and calculation of stresses based on the stress-strain relationship obtained from standard material tensile tests; (**c**) speckle pattern, strain field obtained with DIC, and crack pattern and kinematics computed automatically from the measured DIC strain and deformation fields [221].

**Figure 25:**
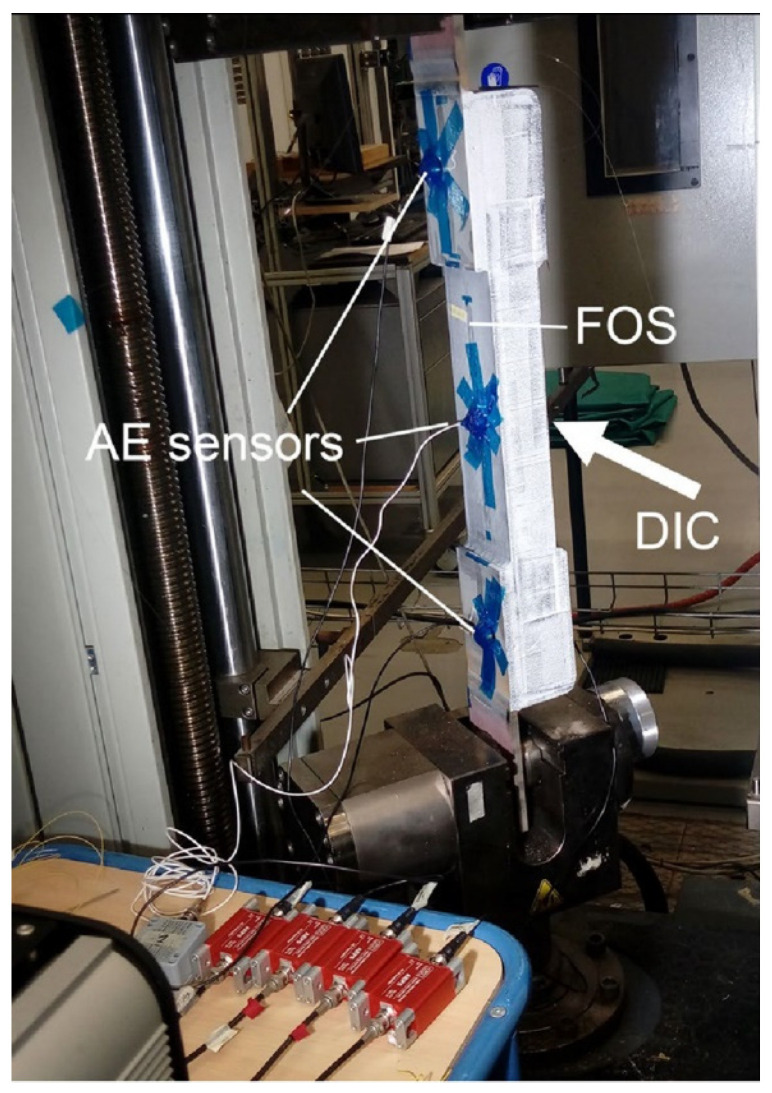
The quasi-static tensile test setup (modified from [260]).

Similarly, Mata-Falcón et al. [221] explored the application of a combination of distributed fiber optic strain measurements on steel reinforcement and DIC measurements on concrete surfaces. They presented the advantages of combining the two measurement techniques: (i) to mutually validate their results, (ii) to relate the stress distribution in the reinforcement to the concrete kinematics, and by this (iii) to investigate the load distribution in the different element parts overtime on a very intuitive basis. Besides that, their main contribution was to discuss the assessment of measurement uncertainty of DIC and FOS in large-scale structural tests. The experimental results showed that the average fiber strain results agreed very well with the DIC results (deviation of about 50 μm/m), and the crack locations predicted by the two measurement methods matched perfectly. The experimental setup they used is shown in Figure 24. Recently, Saeedifar et al. [260] similarly integrated FOS and DIC for SHM of an adhesively bonded bi-material full-scale joint. Their results showed that the measurements obtained by FOS and DIC converged. In addition, they integrated acoustic emission sensing (AE) technology to perform a large number of measurements on structures in the quasi-static tensile test. The setup of corresponding experimental setup is shown in Figure 25. Ultimately, they demonstrated that the integration of the above three sensing technologies can successfully monitor the integrity of adhesively bonded bi-material full-size joints.

## 6. Future Directions of Fiber Optic Sensing Technology and Vision Sensing Technology

From the above-mentioned numerous experimental investigations and engineering application examples, we can see that it is very feasible to apply FOS and vision sensing technology to SHM. They have replaced traditional sensing technology in many fields. However, there are still some directions that need to be studied in depth.

First, there is still always space for breakthrough concepts and ideas that propose radical new solutions. A situation like the proposed concept of fiber optic grating that completely changed the world of fiber optic sensors could come again at any time [151]. For example, new sensors for nonlinear elastic wave spectroscopy (NEWS) [261] and plasmonic optical fiber sensors [262] have been proposed, which may open up more efficient ways to perform SHM.

Second, the measurement range, spatial resolution, and sensitivity of the sensor system need to be further improved. This requires further improvement of the system’s signal reception and processing capabilities, which mainly refers to improvements regarding data processing algorithms.

Third, the long-term stability and service life of the sensor also need to be further improved. For fiber optic sensors, further improvements are needed in their manufacturing process, packaging technology, and installation technology. For vision sensors, more improvements are needed for their vulnerability to environmental factors and hardware device limitations.

Fourth, more research is needed to apply sensing technology to realistic structures. The emerging DFOS technology is still immature, and while there have been some exciting applications, there is still a need for more applications on engineering structures. In addition, some specialized guidance is needed for the test setup and calibration of vision sensing technologies.

Finally, for fiber optic sensing technology, it is difficult to monitor the global displacement of the structure, which can be complemented by vision sensing technology. For vision sensing technology, it is almost impossible to obtain the internal changes of the structure, which can be complemented by fiber optic sensing technology. SHM is a multidisciplinary research project. Thus, one of the promising research directions for the future is the study of various hybrid technologies. Some hybrid fiber optic sensing techniques have been shown above [187], in addition to ultrasonic detection techniques [19], wireless sensing techniques [63], and even machine learning [164] integrated with fiber optic sensing techniques. Likewise, the integration of fiber optic sensing technology and vision sensing technology introduced in this paper is also a very promising direction. The feasibility of integrating these two sensing technologies has been demonstrated by numerous examples in the previous section. For fiber optic sensing techniques, it is possible to obtain the internal changes of a structure, while vision sensing techniques can obtain the external features of the structure. This is necessary for SHM to obtain both changes in the external profile and internal details of the structure. The integration of these two sensing techniques not only helps to mutually verify their measurement uncertainties but also helps to obtain a more comprehensive health status of the structure. Therefore, the next more promising work is to automatically analyze and creatively present the data obtained by these two sensing technologies.

## 7. Conclusions

This paper summarizes the applications and progress of fiber optic and vision sensing technologies for SHM. Fiber optic sensing technologies can be single point, quasi-distributed, and fully distributed, and this paper describes their sensing principles and research progress in detail, giving examples of their applications on various engineering structures. Vision sensing technology has evolved with the advancement of computer technology, and this paper describes its foundations, progress and applications, and summarizes its unique advantages. In addition, this paper discusses the feasibility and potential benefits of integrating these two types of sensing technologies, including mutual verification of measurement reliability, simultaneous information on changes inside and outside the structure, and more accurate safety assessments and future predictions. This integrated approach is relatively recent but can significantly evolve and attract the attention of researchers in the future, especially considering further research to automatically analyze data and creatively present structural state information. Despite some obstacles, we believe that the role of fiber optic and vision sensing technologies in SHM will become increasingly important.

## Figures and Tables

**Table 1:** Comparison of different reflective distributed fiber optic sensing techniques.

Sensing Technology	Key Measurand	Measurement Resolution	Sensing Range	Spatial Resolution	Refs.
Raman OTDR	Temperature	1 °C	25 km	1.13 m	[141]
BOTDR	Temperature and strain	0.93 °C/19.48 µε	10 km	3 m	[142]
BOTDA	Temperature	2 °C	2 km	2 cm	[143]
	Temperature and strain	1.5 °C/30 µε	150 km	2 m	[144]
	Temperature and strain	-	11 km	Sub-cm	[135]
BOCDA	Temperature and strain	-	3 km	2 mm	[133]
OFDR	Temperature and strain	0.8 °C/7 µε	170 m	6.5 mm	[145]
	Temperature and strain	-	20 m	0.17 m	[138]
	Strain	µε-level	140 m	cm-level	[139]
	Strain	-	2 m	2.6 mm	[140]

**Table 2:** The main bridges in the world equipped with SHM systems with fiber optic sensors (modified from [173]).

Bridge Name	Location	Bridge Type	Span (m)	Refs.
Jiangyin Bridge	Jiangsu, China	Suspension	1385	[174]
Sutong Bridge	Jiangsu, China	Cable-stayed	1088	[175]
Stonecutters Bridge	Hong Kong, China	Cable-stayed	1018	[176]
Brooklyn Bridge	NYC, USA	Hybrid (suspension/cable-stayed)	486.3	[177]
I-35W Saint Anthony Falls Bridge	Minneapolis, USA	Box girder	154	[178]
Manhattan Bridge	New York, USA	Suspension	451	[179]

**Table 3:** Comparison of used measurement methods regarding their application ranges [255].

Measurement Method		Extensometers	DIC	DFOS	
				Polyimide	Thorlabs
Strain measurement	Elastic	+	+/-	+	+
	Strain-hardening	+	+/-	-	+
Distributed microcracks	Detection	+	+/-	+	-
	Localization	-	+/-	+	-
	Measurement	-	+/-	+	-
Localized cracks/ fictitious cracks	Detection	+/-	+	+	+
	Localization	-	+	-	+
	Measurement	-	+	-	+
Comments		Limited area covered; simplest in application and analysis	Highly dependent on noise and area of interest	Measurement of microcracks theoretically possible	Crack measurement range limited to 400 µm for UHPFRC

+ ~ yes/- ~ no.

## Data Availability

Not applicable.
